# Culture-negative subacute bacterial endocarditis masquerades as granulomatosis with polyangiitis (Wegener’s granulomatosis) involving both the kidney and lung

**DOI:** 10.1186/1471-2369-13-174

**Published:** 2012-12-26

**Authors:** Hui Peng, Wen-fang Chen, Chao Wu, Yan-ru Chen, Bo Peng, Sujay Dutta Paudel, Tan-qi Lou

**Affiliations:** 1 Division of Nephrology, Department of Medicine, The third affiliated hospital of Sun Yat-sen University, 600 Tianhe Road, Guangzhou, 510630, People’s Republic of China; 2 Zhongshan School of Medicine, Sun Yat-sen University, 74 Zhongshan 2nd Road, Guangzhou, 510080, People’s Republic of China; 3 Department of Pathology, The first affiliated hospital of Sun Yat-sen University, 58 Zhongshan 2nd Road, Guangzhou, 510080, People’s Republic of China

**Keywords:** Subacute bacterial endocarditis, PR3/c-ANCA, Granulomatosis with polyangiitis (Wegener's granulomatosis), Glomerulonephritis

## Abstract

**Background:**

Subacute bacterial endocarditis (SBE) occasionally exhibits positive cytoplasmic anti-neutrophil cytoplasmic antibody (c-ANCA) of the anti-proteinase-3 (PR-3) type. Clinically, it mimics ANCA-associated vasculitis, such as Wegener's disease with glomerulonephritis. Lung abscesses are the most common manifestation of lung involvement. We herein report a case of culture-negative SBE strongly c-ANCA/PR3-positive accompanied by pulmonary involvement and glomerulonephritis. In this case, we took biopsies of both the lung and kidney, although renal biopsy is usually preferred over lung biopsy. The lung biopsy showed severe alveolar capillaritis, suggesting vasculitis consistent with polyangiitis. The renal biopsy revealed glomerulonephritis with a membranoproliferative pattern. To our knowledge, this is the first such reported case.

**Case presentation:**

A 68-year-old Chinese male patient presented to our hospital with a fever, cough, chest pain, and recurrent peripheral edema. He had a past medical history significant for treated schistosomiasis 20 years previously. Physical examination revealed palpable purpura, mild hypertension, hepatosplenomegaly, and a holosystolic cardiac murmur (Levine 2/6). Echocardiography showed tricuspid valve vegetations with moderate to severe regurgitation. Serum c-ANCA/PR3 and cryoglobulin were strongly positive. Renal biopsy results indicated membranoproliferative glomerulonephritis with several crescents. Chest CT revealed multiple intraparenchymal and subpleural nodules, and lung biopsy showed polyangiitis. The patient’s ANCA titers, glomerulonephritis, and pulmonary injury all resolved after antibiotic therapy.

**Conclusion:**

SBE may present with positive c-ANCA/PR3, multiple pulmonary nodules, pulmonary polyangiitis, and glomerulonephritis clinically mimicking granulomatosis with polyangiitis (Wegener's granulomatosis).

## Background

Subacute bacterial endocarditis (SBE) is rarely associated with positivity for anti-neutrophil cytoplasmic/proteinase-3 antibodies (c-ANCA/PR3). SBE may present with a variety of immunologic phenomena, including small vessel vasculitis such as cutaneous purpura, pneumonia, and glomerulonephritis, which may mimic the clinical manifestations of ANCA-associated idiopathic vasculitis with endocardial involvement [1]. Patients with ANCA-associated idiopathic vasculitis more frequently exhibit pulmonary manifestations or pulmonary-renal syndrome. Pulmonary inflammatory granulomas have occasionally been reported in ANCA-positive SBE patients. Their lung injury is most commonly diagnosed as lung abscessation based on the clinical manifestations, and responds to antibiotic treatment without biopsy [2]. However, we herein present a case of a culture-negative SBE patient positive for c-ANCA/PR3 with glomerulonephritis and pulmonary injury. Lung biopsy showed severe inflammation of the alveolar capillary walls. Renal biopsy showed membranoproliferative glomerulonephritis along with crescent formation. To our knowledge, this is the first such reported case. ANCA titers, glomerulonephritis, and pulmonary injury all resolved after a long duration of antibiotic therapy. Such a case poses a challenge for clinicians to differentiate ANCA-positive SBE from ANCA-associated non-infectious endocardial involvement because the treatment strategies for these two conditions are entirely different.

## Case presentation

A 68-year-old male patient was admitted to our hospital in December 2008 with a fever, cough, right-sided chest pain, and swelling of both lower extremities. The patient stated that he had schistosomiasis 20 years previously, which resolved after administration of medication. His medical and family histories were otherwise unremarkable. The patient denied recent dental work and was on no medications at that time. On admission, his body temperature was 38°C and pulse was 105 bpm. Abnormal physical findings included bibasal lung crackles and bilateral lower extremity pitting edema. He had normal heart sounds without a murmur. There were no dermatologic manifestations, nasopharyngeal abnormalities, or inflamed joints. Initial laboratory test results revealed a urine sediment protein of 1+ without blood cells or casts. His hemoglobin was 8.3 mg/dL. His biochemistry profile revealed a normal serum creatinine level and an albumin level of 3.1 mg/dL. Chest X-ray revealed right lower lobe pneumonia with minimal pleural effusion on both sides. He was thus diagnosed with right lower lobe pneumonia with bilateral pleural effusion. Appropriate antibiotic therapy was started immediately, after which the patient improved significantly and was discharged. However, the patient returned to our hospital 1 year later with intermittent bilateral lower extremity edema and skin rashes.

On admission, his vitals were normal with the exception of mild blood pressure elevation (150/80 mmHg). Abnormal physical examination findings included bilateral pitting edema of the lower extremities, extensive purpura on both legs, hepatosplenomegaly, and a holosystolic cardiac murmur (Levine 2/6).

The basic laboratory data were as follows (Table 1): leukocyte count, 3.01 × 10^3^/μL (62.1% neutrophils, 31.5% lymphocytes, 5.5% monocytes, and 0.6% eosinophils); hemoglobin, 5.8 mg/dL (microcytic hypochromic anemia); platelets 59 × 10^3^/μL; total protein, 8.1 g/dL; albumin, 3.1 g/dL; globulin, 5.0 g/dL; serum creatinine, 2.16 mg/dL; and C-reactive protein, 2.62 mg/L. The erythrocyte sedimentation rate was 140 mm/h. Urinary sediment analysis showed protein of 2+ and occult blood of 3+. The rheumatoid factor was 36.2 IU/mL (reference range, <20 IU/mL), and antinuclear antibody was positive with a titer of 1:1000. The serum complement 3 level was low at 32.1 mg/dL (reference range, 80–160 mg/dL), while the total complement and complement 4 levels were within normal limits. Indirect immunofluorescence c-ANCA was positive. The c-ANCA/PR3 titer was significantly elevated at 102 ELISA units (reference range, <15), while p-ANCA/MPO was negative. Viral serology for human immunodeficiency virus, hepatitis B virus, and hepatitis C virus were all unremarkable. A test for cryoglobulin was positive. Serum protein electrophoresis showed increased γ-globulin (48.1%) with mildly increased IgG (4035 mg/dL) and IgM (352 mg/dL).

**Table 1:** Laboratory values of the patients

		**Normal range**	**On admission**	**After treatment**
			**(Dec 2009)**	**(May 2010)**
**Blood**	Hemoglobin (mg/dL)	13.1–17.2	5.8	8.2
	RBC (×10^6^/μL)	4.09–5.74	1.92	2.66
	WBC (×10^3^/μL)	3.97–9.15	3.01	3.20
	PLT (×10^3^/μL)	100–300	59	111
**Urine**	Protein	Negative	(++)	(-)
	Occult blood	Negative	(+++)	(+)
	RBC (count/μL)	0–18	48	8
**Serum**	Albumin (g/dL)	3.60–5.10	3.10	3.98
	Globulin (g/dL)	2.50–3.50	5.00	3.83
	Creatinine (mg/dL)	0.42–1.52	2.16	1.35
	CRP (mg/L)	0–6	2.62	0.95
	C3 (mg/dL)	80–160	32.10	91.3
	Cryoglobulin	Negative	(+)	(-)
**Immunological indicators**	RF (IU/mL)	<20	36.2	(-)
	ANA	Negative	1:1000	(-)
	C-ANCA	Negative	(+)	(-)
	PR3-ANCA (U/mL)	<15	102	(-)

RBC: red blood cells, WBC: white blood cells, PLT: platelets, CRP: C-reactive protein, C3: complement C3, RF: rheumatoid factor, ANA: autoantibody to nuclear antigen, C-ANCA: cytoplasmic anti-neutrophil cytoplasmic antibody, PR3-ANCA: proteinase 3 anti-neutrophil cytoplasmic antibodies, (+): positive, (-): negative or not detectable.

**Figure 1:**
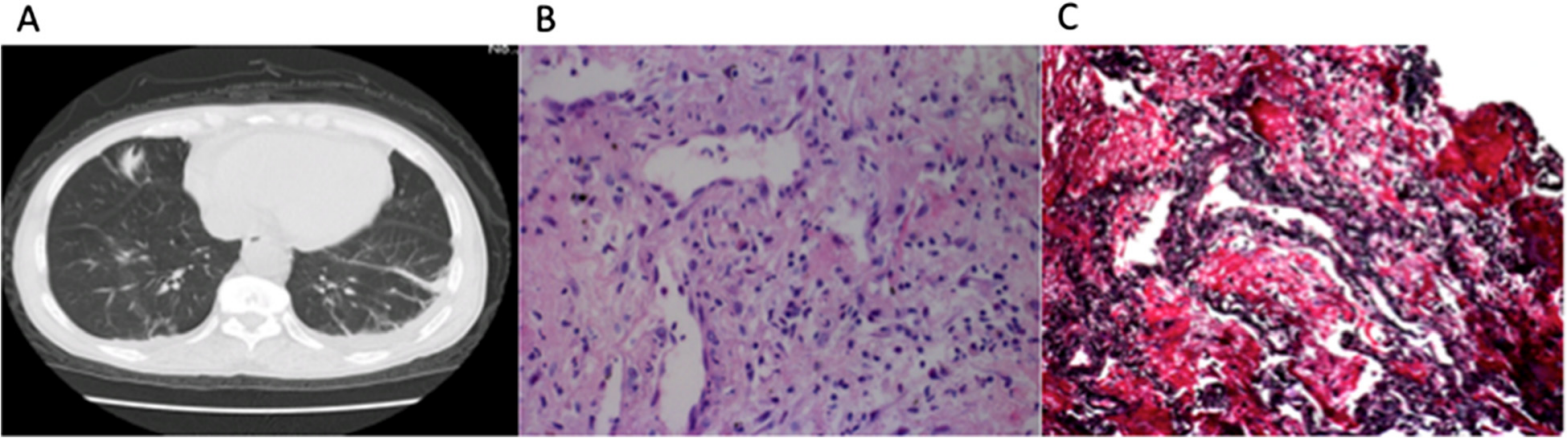
**A) Chest CT showing a solid intraparenchymal nodule in the right lung with enlargement of hilar lymph nodes and presence of pleuritis with minimal bilateral pleural effusion.B**) Lung biopsy showing alveolar epithelial cells falling off and a large amount of fibrin in the alveolar cavity. Neutrophils and a few lymphocytes are infiltrating the interstitium (H&E stain, original magnification x400). **C**) Lung biopsy showing broken capillary walls of the alveoli. A large amount of fibrin is seen in the alveolar cavities (PASM & Masson, original magnification ×400).

Chest CT showed multiple intraparenchymal and subpleural nodules on both lungs (solid or with a ground-glass character), hilar lymphadenopathy with pleural thickening, and minimal bilateral pleural effusion (Figure 1A). Fiberbronchoscopic biopsy was performed, and PASM staining showed no granulomas, but there was severe alveolar capillaritis. The alveolar epithelial cells were detached, and a large amount of fibrin filled the alveolar cavity. There were numerous neutrophils and a few lymphocytes infiltrating the interstitium (Figure 1B,C). The pulmonary inflammation resembled polyangiitis. The histology was compatible with the criteria of granulomatosis with polyangiitis (GPA) according to the Chapel Hill Consensus Criteria (1992), although granulomas were not found, possibly due to sampling error. Thus, the histopathological findings, chest CT results, and lung manifestations all mimicked GPA (Wegener’s granulomatosis).

**Figure 2:**
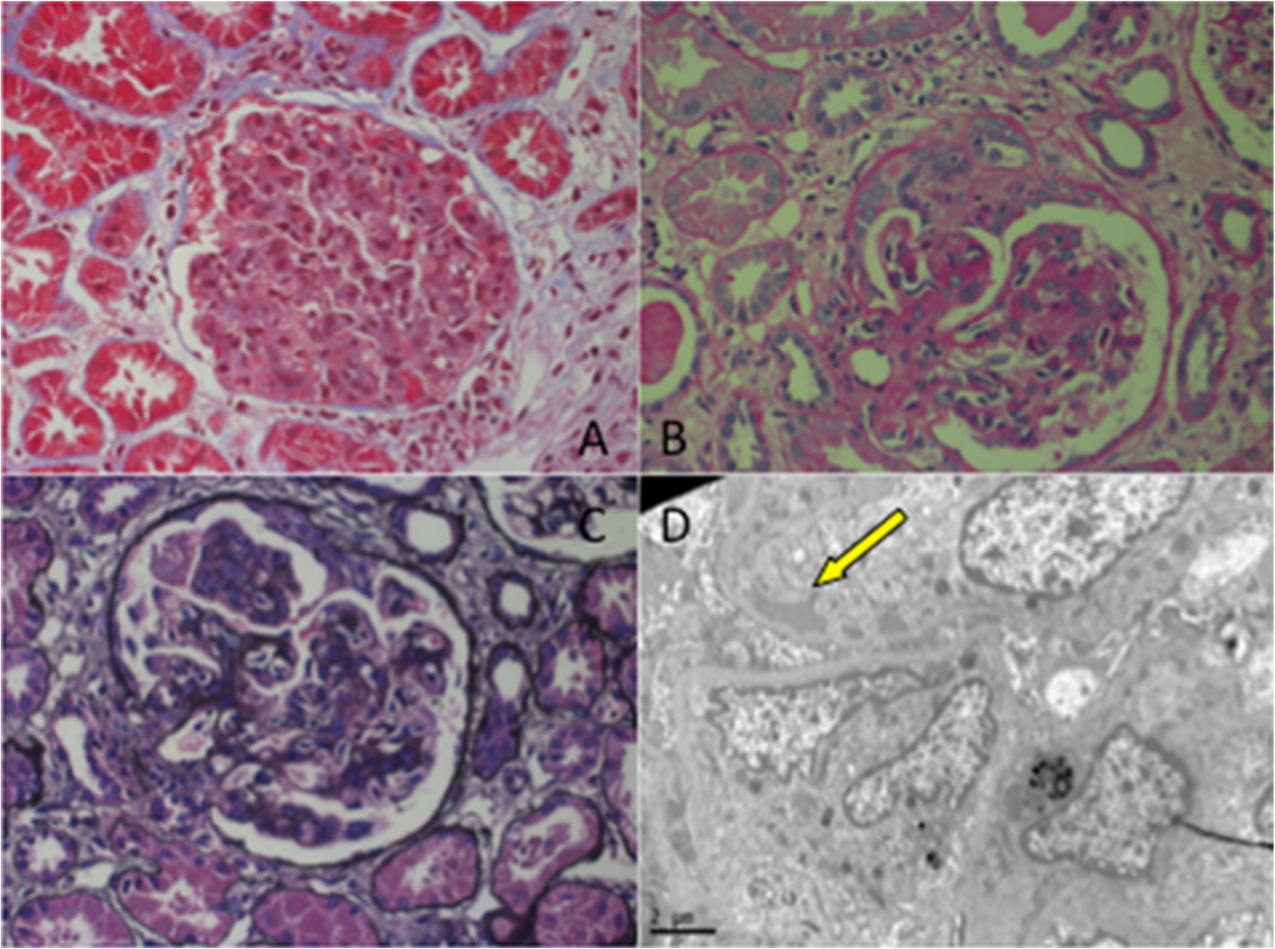
**A) Glomerular mesangial and endothelial cell proliferation (Masson’s trichrome; original magnification ×400).B**). Small cellular crescent formation in several glomeruli (Periodic acid-Schiff; original magnification ×400). **C**) Subendothelial deposits (Masson’s trichrome and Jones methanamine silver; original magnification ×400). **D**) Glomerular hypercellularity with dense deposits (arrow) in the subendothelial area (electron microscopy; original magnification ×2000).

Because ANCA-associated rapidly progressive glomerulonephritis was suspected, a renal biopsy was performed. The result revealed diffuse proliferation of mesangial and endothelial cells accompanied by several crescents, segmental interposition of the mesangium, and occasional thrombi in the capillary loops (Figure 2A,B,C). Immunofluorescence staining showed spotty granular staining of IgG (2+), IgM (3+), C3 (3+), C1q (2+), and Fg (2+) along the peripheral capillary walls. Electron micrographs of capillary walls from a glomerulus showed dense deposits in the subendothelial area with endocapillary hypercellularity (Figure 2D). The renal damage was therefore not consistent with GPA or ANCA-associated vasculitis, but rather suggestive of cryoglobulinemia-induced renal damage [3].

Five days after admission, the patient spiked a fever of 39°C with chills, nausea, and decreased appetite. Echocardiography revealed tricuspid valve vegetations with moderate to severe regurgitation. Aerobic and anaerobic blood cultures were obtained before the initiation of antimicrobial therapy, and the results showed no growth of microorganisms. Repeat cultures performed after the initiation of antibiotic therapy remained negative. Based on the echocardiographic findings and the clinical course, culture-negative SBE of the tricuspid valve was strongly suspected, and intravenous cefoxitin was initiated for a duration of 10 days as empirical antimicrobial therapy. The patient’s temperature returned to normal after 5 days of treatment. Seven days thereafter, both the edema and purpura subsided. There was an increase in his appetite and a sense of well-being. For SBE, cefoperazone and tazobactam were prescribed for duration of 3 months. During the course of antibiotic therapy, repeated chest X-ray showed remarkable improvement with resolution of the pulmonary nodules. Most of the laboratory test results reverted back to normal after 3 months of treatment (serum globulin, 3.83 g/dL; albumin, 3.98 g/dL; IgG, 2.86 g/dL; serum creatinine, 1.35 mg/dL; leukocyte count, 3.20 × 10^3^/μL; hemoglobin, 8.2 mg/dL; platelets, 111 × 10^3^/μL). Urinary protein was negative with occult blood of 1+. ANA and RF were both negative. Cryoglobulin returned to negative. The C-ANCA/PR3 titer decreased from 125 IU/mL (highest) to negative (see Table 1). The patient thus remained asymptomatic with no signs of fever, edema, or purpura, and there were no complaints of nausea or fatigue.

## Discussion

We reported an elderly male patient with culture-negative SBE associated with strongly positive c-ANCA/PR3. His clinical manifestation masqueraded as GPA with pulmonary nodules and glomerulonephritis. Renal biopsy showed glomerulonephritis with a membranoproliferative pattern along with several crescents. Lung biopsy showed severe alveolar capillaritis resembling polyangiitis. We believe that this is the first such reported case of both lung and renal involvement confirmed by laboratory, imaging, and biopsy results. His glomerulonephritis, pulmonary lesions, and elevated c-ANCA resolved after a course of antibiotic therapy.

Proteinase-3/c-ANCA (PR3/C-ANCA) is highly associated with GPA, with a sensitivity and specificity of >90%. There may be exclusive cases in which PR3/C-ANCA tests are positive in patients with SBE, as reported in our case [4]. It has been suggested that the induction of ANCA in infectious diseases, more specifically in SBE, may occur through nonspecific B-cell activation or autoimmunization after the release of PR3 or MPO from neutrophils, and PR3/C-ANCA is the most common ANCA type [5]. The details of the underlying mechanism remain unknown. Some clinical features of GPA and infective endocarditis overlap, including vegetations detectable by echocardiography, inflammatory signs, and renal involvement with constitutional symptoms [6]. Necrotizing granulomatous inflammation of the respiratory tract is one of the characteristic pathological features of GPA. Although valvular involvement has been described in sporadic case reports of GPA [7], granulomatous inflammation of the lung is not common in SBE in ANCA-positive patients. A total of 23 cases of ANCA-positive SBE have been described in the literature to date [2-9]. Organ involvement in SBE patients is usually limited to the skin and kidneys. This is considered to be one of the differentiating features indicative of SBE rather than GPA. A few cases of lung involvement in SBE have been reported. Lung injury is usually diagnosed as lung abscessation based on the clinical manifestations and a positive response to antibiotic therapy [2,10]. However, we must be aware that the lung may also be involved in SBE patients with positive ANCA, showing pathological changes indicative of alveolar capillaritis, as seen in our case. We cannot rule out the presence of granulomatous lung lesions in this case due to possible sampling error during the biopsy. However, we confirmed that no lung abscess was present; however, ANCA-associated vasculitis-like lung injury was identified, based on the CT scan results and lung pathology report.

Although the renal and lung manifestations are similar to those of GPA, SBE may also manifest with positive ANCA and multiple serological abnormalities [2]. The following characteristics—described by Chirinos *et al.*[5]—are pertinent differentiating features and were found to be more indicative of SBE than GPA: splenomegaly, hypocomplementemia, elevated RF, cryoglobulinemia, positive antinuclear and anticardiolipin antibodies, and positive blood cultures. A negative blood culture is a diagnostic and therapeutic challenge for physicians. In our case, one possible reason for the negative blood culture may have been the antibiotic treatment that preceded the collection of the blood specimen. Another possibility is a specific bacterial infection that grows only slowly. This case fulfilled one major (oscillating intracardiac mass on echocardiogram) and two minor (fever of >38°C and immunologic phenomena) Duke’s criteria. Although they were not diagnostic, these findings were highly suggestive of a clinical working diagnosis of SBE and prompted antibiotic treatment. We believe that without convincing evidence of GPA, SBE cannot be ruled out and immunosuppressive therapy should not be initiated. Immunosuppressive treatment of this condition may aggravate the infection and have life-threatening consequences.

## Conclusion

This case involved SBE with PR3/C-ANCA, membranoproliferative glomerulonephritis, multiple lung nodules, and pulmonary polyangiitis that clinically mimicked GPA with many overlapping clinical manifestations. This is the first case to show pathologic involvement of both the kidney and lung. It is therefore important to recognize the possibility of coexisting bacterial endocarditis to achieve the correct diagnosis.

## Consent

Informed written consent was obtained from the patient for publication of this case report and any accompanying images. A copy of the written consent is available for review by the Editor-in-Chief of this journal.

## Abbreviations

GPA: Granumatosis with Polyangiitis (*Wegener's*); SBE: Subacute bacterial endocarditis; PR3/C-ANCA: Proteinase-3/cytoplasmic antineutrophil cytoplasmic antibodies; SVV: Small vessel vasculitis; AAV: ANCA-associated idiopathic vasculitis; ESR: Erythrocyte sedimentation rate.

## Competing interests

The authors declare that they have no conflicts of interests.

## Authors’ contributions

HP, WC and CW were involved in acquiring data, conception, design and writing the manuscript; WC was involved in pathology data, and YC, BP and SDP were involved in patient care and manuscript preparation; TL were involved in patient care, conception, design, drafting and revising the manuscript. All authors have read and approved the final manuscript.

## Pre-publication history

The pre-publication history for this paper can be accessed here:

http://www.biomedcentral.com/1471-2369/13/174/prepub

## References

[B1] Subra JF Michelet C Laporte J Carrere F Reboul P Cartier F Saint-André JP Chevailler A The presence of cytoplasmic antineutrophil cytoplasmic antibodies (C-ANCA) in the course of subacute bacterial endocarditis with glomerular involvement, coincidence or association? Clin Nephrol 1998 49 15 18 9491280

[B2] Bonaci-Nikolic B Andrejevic S Pavlovic M Dimcic Z Ivanovic B Nikolic M Prolonged infections associated with antineutrophil cytoplasmic antibodies specific to proteinase 3 and myeloperoxidase: diagnostic and therapeutic challenge Clin Rheumatol 2010 29 893 904 10.1007/s10067-010-1424-4 20306213

[B3] Muller Kobold AC van der Geld YM Limburg PC Tervaert JW Kallenberg CG Pathophysiology of ANCA-associated glomerulonephritis Nephrol Dial Transplant 1999 14 1366 1375 10.1093/ndt/14.6.1366 10382995

[B4] Choi HK Lamprecht P Niles JL Gross WL Merkel PA Subacute bacterial endocarditis with positive cytoplasmic antineutrophil cytoplasmic antibodies and anti-proteinase 3 antibodies Arthritis Rheum 2000 43 226 231 10.1002/1529-0131(200001)43:1<226::AID-ANR27>3.0.CO;2-Q 10643719

[B5] Tiliakos AM Tiliakos NA Dual ANCA Positivity in Subacute Bacterial Endocarditis J Clin Rheumatol 2008 14 38 40 10.1097/RHU.0b013e318164187a 18431098

[B6] Chirinos JA Corrales-Medina VF Garcia S Lichtstein DM Bisno AL Chakko S Endocarditis associated with antineutrophil cytoplasmic antibodies: a case report and review of the literature Clin Rheumatol 2007 26 590 595 10.1007/s10067-005-0176-z 16440133

[B7] Goodfield NE Bhandari S Plant WD Morley-Davies A Sutherland GR Cardiac involvement in Wegener's granulomatosis Br Heart J 1995 73 110 115 10.1136/hrt.73.2.110 7696016 PMC483773

[B8] Neumann I Regele H Kain R Birck R Meisl FT Glomerular immune deposits are associated with increased proteinuria in patients with ANCA-associated crescentic nephritis Nephrol Dial Transplant 2003 18 524 531 10.1093/ndt/18.3.524 12584274

[B9] Messiaen T Lefebvre C Zech F Cosyns JP Jadoul M ANCA-positive rapidly progressive glomerulonephritis: there may be more to the diagnosis than you think! Nephrol Dial Transplant 1997 12 839 841 10.1093/ndt/12.4.839 9141032

[B10] Uh M McCormick IA Kelsall JT Positive Cytoplasmic antineutrophil cytoplasmic antigen with PR3 specificity glomerulonephritis in a patient with subacute bacterial endocarditis J Rheumatol 2011 38 1527 1528 21724732 10.3899/jrheum.101322

